# Toxicity, Tunneling and Feeding Behavior of the Termite, *Coptotermes vastator*, in Sand Treated with Oil of the Physic Nut, *Jatropha curcas*

**DOI:** 10.1673/031.009.6401

**Published:** 2009-12-03

**Authors:** Menandro N. Acda

**Affiliations:** Dept. of Forest Products and Paper Science, College of Forestry and Natural Resources, University of the Philippines Los Banos, College, Laguna 4031, Philippines

**Keywords:** phorbol esters, curcin, repellency, sand barrier

## Abstract

Oil of the physic nut, *Jatropha curcas* L. (Malpighiales: Euphorbiaceae), was evaluated in the laboratory for its barrier and repellent activity against the Philippine milk termite *Coptotermes vastator* Light (Isoptera: Rhinotermitidae). The study showed that *J. curcas* oil had anti-feeding effect, induced reduction in tunneling activity and increased mortality in *C. vastator*. Behavior of termites exposed to sand treated with *J. curcas* oil indicated that it is toxic or repellent to *C. vastator*. Toxicity and repellent thresholds, were higher than those reported for other naturally occurring compounds tested against the Formosan subterranean termite.

## Introduction

The physic nut, *Jatropha curcas* L. (Malpighiales: Euphorbiaceae), also known as the Barbados nut or purging nut, is a small, deciduous tree cultivated in Central and South America, Southeast Asia and Africa primarily as a living fence to protect fields (Schmook and Seralta-Peraza 1997). *J. curcas* grows well on marginal to poor soil and can easily be propagated from cuttings or seeds (Heller 1996). It is highly resistant to drought and often used for prevention of soil erosion or planted to reclaim eroded areas in arid and semi-arid tropical and subtropical regions of the world (Heller 1996; Wiesenhütter 2003). Many parts of the plants are used in various tropical countries for treatment of a variety of ailments (Duke 1985), soap production (Henning 1994) and manufacture of organic fertilizer (Sherchan et al. 1989). The seed of *J. curcas* is a good source of fuel for cooking, lighting and diesel engines (Ishii and Takeuchi 1987). The latter use has implications as a practical substitute for fossil fuel and income generation in rural areas of developing countries. The seeds of *J. curcas*, however, are toxic to humans, rodents, snails and livestock (Adam 1974; Adam and Magzoub 1975; Stirpe et al. 1976; Ahmed and Adam 1979a, 1979b; Joubert et al. 1984; Jadhau and Jadhua 1984; Liu et al. 1997; Rug et al. 1997; Rug and Ruppel 2000; Adebowale and Adedire 2006).

Extracts and crude oil from the seeds of *J. curcas* have traditionally been used as an insect repellent, molluscide and rodenticide (Duke 1985). Toxicity of *J. curcas* seeds is attributed to several components, including saponins, lectin (curcin), phytates, protease inhibitors, and curcalonic acid and phorbol esters (Adolf et al., 1985, Makkar et al. 1997, Martinez-Herrera et al. 2004)). However, reports have identified phorbol esters as the main toxic agent responsible for the insecticidal and mollucicidal activities of *J. curcas* oil (Makkar and Becker 1997a, 1997b; Liu et al. 1997). Phorbol esters are tetracyclic diterpenoids that mimic the action of diacyl glycerol, activator of protein kinase C that interferes with different signal transduction pathways and other cellular metabolic activities (Bershadsky et al 1990; Goel et al. 2007). Phorbol esters are also known purgative, skin irritant and tumor promoters but are not mutagenic or carcinogenic themselves (Adolf et al. 1984; Hirota et al. 1988).

The insecticidal activity of *J. curcas* oil containing phorbol esters have been reported for the tobacco hornworm, *Manduca sexta*, cotton bollworm, *Helicoverpa armigera*, melon aphid, *Aphis gossypii*, pink bollworm, *Pectinophora gossypiella*, leafhoppers, *Empoasca biguttula*, Chinese bean weevil, *Callosobruschus chinensis*, maize weevil, *Sitophilus zeamays*, potato tuberworm moth, *Phthorimaea operculella*, pink stalk borer, *Sesamia calamistis*, American cockroach, *Penplaneta Americana*, German cockroach, *Blatella germanica* and the milkweed bug, *Oncopeltus fasciatus* (Shelke et al. 1985; Sauerwein et al. 1993; Solsoloy 1995; Wink et al. 1999).

However, its activity has not yet been evaluated against subterranean termites. This paper reports on the termiticidal activity of *J. curcas* oil against the Philippine milk termite (*Coptotermes* *vastator* Light) (Isoptera: Rhinotermitidae), a major structural pest of timber and wood products in the Philippines. *C. vastator* has been reported to be synonymous with *C. gestroi*, an economically important termite species in Malaysia and other Asian countries (Yeap et al. 2007).

## Materials and Methods

### Termites

Secondary nests of three active field colonies of *C. vastator* were collected from the grounds of the University of the Philippines Los Banos campus and placed in black garbage bags. The nests were immediately transported to the laboratory and placed inside 100 liter plastic containers with lids and kept in a room at 25° C for three days. Distilled water was sprayed on the sides of the container to keep the relative humidity above 80%. Mature worker (pseudergates beyond the third instar as determined by size) and soldier termites were separated from nest debris by breaking apart and sharply tapping materials into plastic trays containing moist paper towels. Termites were then sorted using a soft bird feather and used for bioassay within one hour of extraction and segregation.

### *Jatropha curcas* oil

Mature *J. curcas* seeds were obtained from wild varieties growing in farms in the province of Laguna and identity verified at the College of Forestry and Natural Resources, University of the Philippines Los Banos. The seeds were cracked, the shells carefully removed and the kernels were used for oil extraction. The kernels were wrapped in cheese cloth and pressed using a laboratory Carver press to extract the crude oil. The crude oil suspension was then centrifuged at 10,000 rpm for 20 minutes and the oil phase was collected. The *J. curcas* oil had a specific gravity of 0.90 at 25° C as determined using the pycnometer method (AOAC 1980) and had a clear to light yellow color.

### Sand barrier bioassay

Three-compartment glass containers with lids similar to those described by Maistrello et al. (2001a), with some modifications, were used to test if sand treated with crude *J. curcas* oil will prevent termites from tunneling across the barrier and reach the food source. Glass boxes (18 × 6 × 6 cm) divided into three equal chambers (6 × 6 × 6 cm) were used in this study. The three compartments of the container were named (1) the untreated chamber containing 50 grams untreated sand which is where termites were placed initially (2) the treated chamber (the sand barrier), in the middle containing 50 grams of sand treated with *J. curcas* oiland (3) a food chamber containing 50 grams untreated sand and a piece of filter paper (1.5 × 1.5 cm, Whatman #1) placed on the sand to serve as a food source. Five concentrations of the oil (0, 2.5, 5.0, 10 and 20 % (w/w) were tested in this study. Sand moistened with distilled water was used as control. Small openings (3 × 0.3 cm) at the bottom of each of the two inner walls connecting the chambers were made to allow termites to cross from the untreated chamber to the food chamber. Two hundred worker termites plus twenty soldiers were placed in the untreated chamber. The experimental units were held in an incubator maintained at 28° C and at least 80% relative humidity. Paper consumption and the numbers of live termites in each chamber were recorded after 14 days. The number of dead termites was calculated by substracting the number of live termites from the total number placed in each unit. The bottoms of each container were scanned (Scanjet 2300c, Hewlett Packard, www.hp.com) to fix the configuration and image of the tunnels. Termite behavior and tunneling activities were scanned regularly. Tunneling activities in all chambers were qualitatively scored by three individuals using a scale from 0 to 4; 0 for no tunneling activity, 1 for tunneling activities ≤25% of total chamber area, 2 for 26–50% of total chamber area, 3 for 51–75% of total chamber area and 4 for tunneling activities ≥75% of total chamber area. The assay was performed for three colonies and replicated three times. Differences in tunneling activity, paper consumption and mortality of termites were analyzed for each colony and between colonies using a completely randomized design. The same variables were compared between colonies using randomized complete block design with colony origin a blocking factor (Statgraphics 1999). Significant differences in tunneling activity, paper consumption and mortality were separated by Tukey's Honest Significant Difference (HSD) test at α = 0.05.

### Choice assay of treated and untreated sand

Three-chambered glass containers containing sand similar to that described above were used to test repellency of *J. curcas* oil to *C. vastator*. The middle chamber contained untreated moist sand. One lateral chamber contained 50 grams of untreated sand with moist filter paper (1.5 × 1.5 cm, Whatman #1) placed on top to serve as food and the other lateral chamber 50 grams of sand treated with *J. curcas* oil with moist filter paper on top. Five concentrations, 0, 2.5, 5.0 10.0 and 20.0 % (w/w), were used for these tests. Two hundred worker termites plus twenty soldiers were released in the middle chamber. Termites were allowed to choose between treated and untreated chambers. The experimental units were held in an incubator and the numbers of live termites in each chamber were recorded after 14 days as described earlier. Mortality, paper consumption, termite distribution in each chamber, tunneling pattern and behavior were monitored regularly as described above. Termite distribution or abundance was calculated as the ratio of the number of live termites in each chamber to the total number of termites released in the unit. Significant differences in mortality, paper consumption and termite distribution were statistically analyzed as described above.

## Results and Discussion

### Sand Barrier Assay

**Figure 1.**
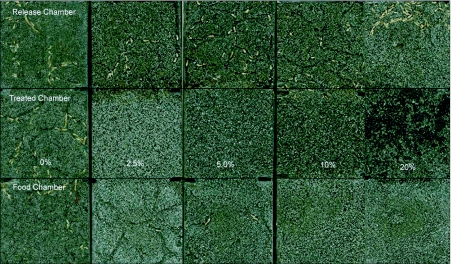
Tunneling characteristics of *Coptotermes vastator* after exposure to sand treated with *Jatropha curcas* oil for 14 days. The glass containers are divided into an untreated chamber (above) containing untreated sand and where termites were initially released, a middle chamber containing sand treated with *J. curcas* oil and a food chamber (bottom) containing untreated sand and filter paper.

Sand treated with *J. curcas* oil showed highly significant effects on tunneling activity, paper consumption and mortality of *C. vastator* (Table 1). The effects of the termite colony used were not significant (*P* > 0.119). Termites were able to construct tunnels and crossed the sand barrier (6 cm) treated with 0, 2.5 and 5% *J. curcas* oil within 24 to 48 hours of exposure. But only control units showed elaborate tunneling in all chambers (Figure 1). Termites did not construct tunnels in chambers containing sand treated with 2.5 and 5% oil but entered the treated area to reach the food chamber. Our scanning observations indicated that termites crawled on the surface of the treated sand to reach the paper in the food chamber. This suggests that *C. vastator* was tolerant of at least 5% *J. curcas* oil but contact with the oil may have some effects on feeding or mortality of termites. *C. vastator* that crawled over sand treated with 2.5 or 5% oil showed higher mortalities (>21%) and reduced paper consumptions than that of the control (Table 1). It is possible that the termites were exposed to toxic components in *J. curcas* oil that produced sublethal effects such as lethargy or paralysis. Components of *J. curcas* oil, possibly phorbol ester or curcin, may have toxic effects on *C. vastator*. Phorbol esters are known to interfere with the normal functions of the metabolic activities of insects (Goel et al. 2007). Unsaponifiable matter such as sterols and terpenes in the oil may also have anti-feeding effects on *C. vastator* similar to those reported for some stored products pests (Makanjoula 1989; Shukla et al 1996; Jagdeesh et al. 1998; Uppuluri et al. 2003).

Containers with sand treated with 10 and 20% *J. curcas* oil prevented termites from breaching the barrier during the 14 days exposure period. Termites remained in the untreated chambers, either close to the border of sand treated with 10% oil or in the far corner of sand treated with 20% oil (Figure 1). No tunnels were made in the treated or food chambers in all units containing sand treated with 10 and 20 % *J. curcas* oil. A rapid increase in mortality (>80%) was observed in units containing sand treated with 10 and 20% oil after about 7 days of exposure. Apparently, termites died of starvation due to their inability to cross the treated area to gain access to the food chamber. Temperate and subtropical termites have been shown to live for at least 10–12 days without food with no appreciable effect on mortality (Su and La Fage 1986; Ibrahim et al. 2004a). However, tropical species such as *C. vastator* seemed viable only for about one week under laboratory conditions without food. Comparison of toxic threshold or repellent activity of *J. curcas* oil against *C. vastator* showed that it is higher than those reported for other long chain oils (Maistrello et al. 2001a, 2001b; Zhu et al. 2001) and from short chain essential oils and other plant extracts (Serit et al. 1992; Grace and Yates 1992, Cornelius 1997; Supriana 1998, Ibrahim et al. 2004b, Osbrink et al 2005, Mao and Henderson 2007, Raina et al. 2007). Moreover, the effect of *J. curcas* oil did not manifest itself immediately after contact but rather slowly within one week of exposure. The nature of this effect is not yet clear but it is possible that *J. curcas* oil breaks up the termite's cuticle or the waxy layer covering their integument similar to those observed with insecticidal soaps. Similar effects on mortality and feeding behavior were observed with of *Coptotermes formosanus* when exposed to nootkatone and vetiver oil (Maistrello et al. 2001a) or acetonaphthone (Ibrahim et al. 2004b).

**Table 1.**
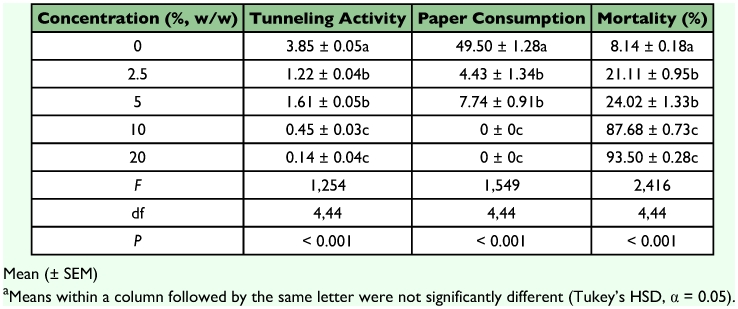
Tunneling, feeding, and mortality of *C. vastator* after exposure to sand treated with *J. curcas* oil for 14 days^a^.

### Choice assay of treated and untreated sand

**Figure 2.**
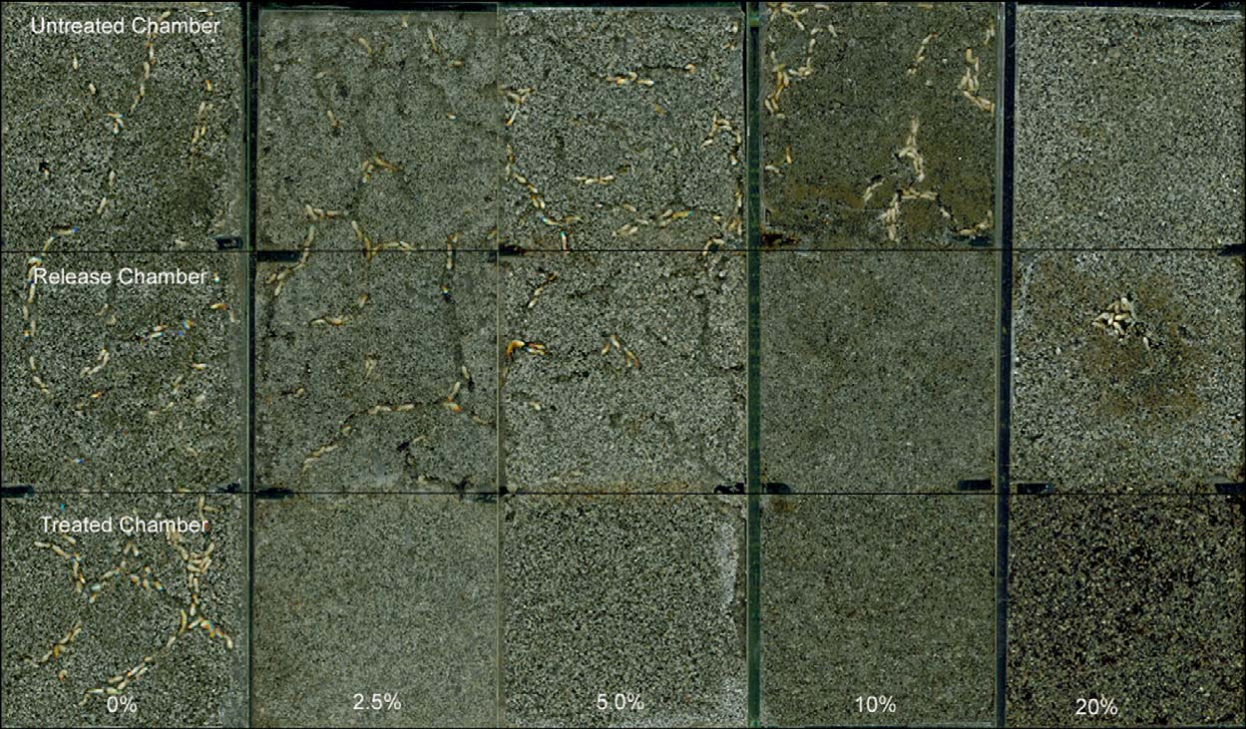
Tunnel patterns during choice feeding assay of *Coptotermes vastator* in sand treated with *Jatropha curcas* oil for 14 days. The glass containers are divided into an untreated chamber containing untreated sand and filter paper (above), a middle chamber containing untreated sand where termites were initially released and a treated chamber (bottom) containing sand treated with *J. curcas* oil and filter paper.

In choice tests, chambers containing treated and untreated sand showed significant differences in termite distribution after 14 days of exposure (*P* < 0.001, Table 2). Except for the control units, most of the termites remained in the middle chamber with untreated sand or in the chamber with untreated sand and paper in all concentrations tested. Occasionally, some termites were observed exploring the chamber with sand treated with 2.5 or 5% oil and signs of nibbling were present on paper placed on top of these units. Apparently the presence of *J. curcas* oil disrupted termite behavior and elicited a repellency response away from the treated sand (Figure 2). In sand treated with 20% oil, termites remained clumped together in the bottom of the middle chamber in a cavelike structure. Similar “lingering behavior” was observed in *C. formosanus* by Maistrello et al. (2001a) with sand treated with nootkatone or vetiver oil. The reason for this unusual behavior is still unclear but it is possible that this may be a response to some degree of volatization of the oil from the treated chamber. Comparison of paper consumption in treated and untreated chambers also indicated low feeding rate in units containing *J. curcas* oil as compared to that of the control units. This suggests an anti-feeding effect of the oil on *C. vastator* even if termites were not in the treated area (Table 3).

**Table 2.**
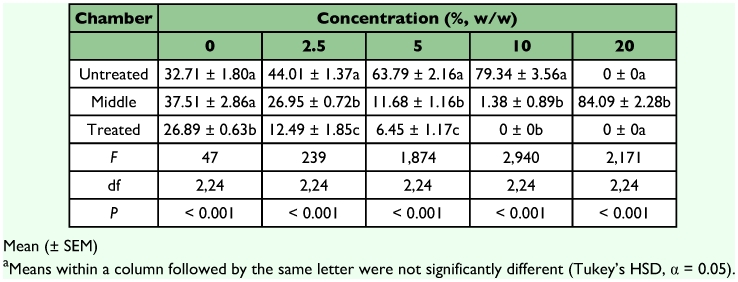
Percent distribution (%) of live *C. vastator* in glass chambers containing sand either treated or untreated with *J. curcas* oil after 14 days^a^.

In general, *J. curcas* oil of at least 10% concentration proved to be a toxic or repellent barrier for *C. vastator*. Lower doses of *J. curcas* oil induced reduction in tunneling activity, paper consumption and a small increased mortality. The toxicity and repellent thresholds of *J. curcas* oil against *C. vastator* reported in this study was much higher than those reported for other naturally occurring compounds previously tested against the Formosan subterranean termite, *C. formosanus*. This could be problematic considering that potential application would require a large volume of oil to control tunneling and feeding of *C. vastator*. Alternatively, the oil could be used treat wood to prevent termite attack. Despite the low toxicity to *C. vastator, J. curcas* oil remains an attractive anti-termitic agent due to the large sustainable supply of the material from Asia and Africa. In addition, a significant increase in termite activity may be achieved by enhancing its toxic components through extraction using organic solvents. Enhancing the amount of phorbor esters or curcin extracted using methanol or acetone showed significant improvement in insecticidal and molluscicidal activities compared to crude *J. curcas* extracts (Liu et al. 1997; Rug et al. 1997).

**Table 3.**
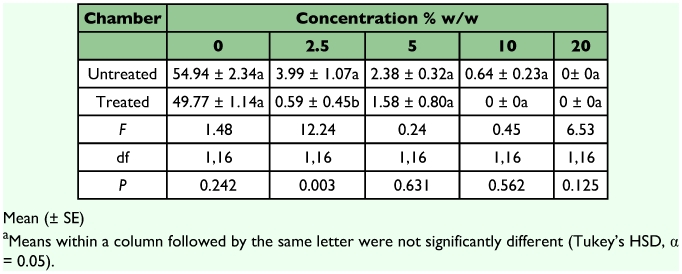
Paper consumption (mg) by *C. vastator* over a 14 day period in treated and untreated chambers of the choice feeding assay^a^.
